# Altered power spectral density in the resting-state sensorimotor network in patients with myotonic dystrophy type 1

**DOI:** 10.1038/s41598-018-19217-0

**Published:** 2018-01-17

**Authors:** Jin-Sung Park, Jeehye Seo, Hyunsil Cha, Hui-Jin Song, Sang-Hoon Lee, Kyung Eun Jang, Hui Joong Lee, Juyoung Park, Ho-Won Lee, Yongmin Chang

**Affiliations:** 1 Department of Neurology, Kyungpook National University School of Medicine, Daegu, Korea; 2 Department of Medical & Biological Engineering, Kyungpook National University, Daegu, Korea; 3 Institute of Biomedical Engineering Research, Kyungpook National University, Daegu, Korea; 4 Department of Radiology, Kyungpook National University School of Medicine, Daegu, Korea; 5 Daegu-Gyeongbuk Medical Innovation Foundation, Medical Device Development Center, Daegu, Korea; 6 Department of Molecular Medicine, Kyungpook National University School of Medicine, Daegu, Korea

## Abstract

Myotonic dystrophy type 1 (DM1) is a multisystemic disease that involves the brain with several neurological symptoms. Although there were few imaging studies on DM1, no studies have investigated functional alterations in the sensorimotor network at rest in patients with DM1. In the current study, a power spectral density (PSD) analysis of resting-state fMRI data was performed to assess possible alteration in spontaneous neural activity of the sensorimotor network in patients with DM1. Compared to healthy controls, patients with DM1 showed higher PSD responses in the orbitofrontal cortex, parahippocampus and basal ganglia (corrected *P* < 0.05). Patients with DM1 showed higher PSD responses in white matter structures associated with motor function (corrected *P* < 0.05). Furthermore, correlation analysis indicated that the brain regions showing PSD differences were correlated with measures of motor performance (*P* < 0.05). In gray matter, our findings suggest that motor disability in DM1 is not an isolated deterioration of the motor power but a multimodal dysfunction that also involves the visual system. In addition, the widespread PSD alteration in white matter structures suggest that motor deficits in DM1 involve motor movement structures as well as structures important for its coordination and regulation.

## Introduction

Myotonic dystrophy type 1 (DM1) is the most common type of muscular dystrophy in adults^1^. DM1 is an autosomal dominantly inherited multisystemic disease that involves the brain, and several neurological symptoms have been associated with DM1^2,3^. DM1 is clinically characterized by predominant motor weakness in the facial, long finger flexors, and feet dorsiflexors. Additionally, it is associated with delay in fully extending the fingers after forceful hand contraction, which is known as grip myotonia.

Brain involvement in DM1 has been well described with various neuroimaging techniques^4,5^. Neuroimaging studies have shown a wide range of structural brain abnormalities in DM1, most importantly white matter disease and atrophy. White matter abnormalities are detected throughout the whole brain in DM1, including in the association fibers, commissural fibers, and projection fibers that connect the several cortical areas with the brain^6–10^. Although less pronounced than that in white matter, widespread gray matter atrophy is observed in the frontal, parietal, temporal, and occipital regions. Subcortical gray matter atrophy is also detected in the thalamic and basal ganglia structures^10–12^. However, the potential correlation between neuroimaging alterations and clinical/genetic factors such as CTG repeat length in the altered brain regions remains unclear.

Recently, a few studies revealed functional alterations in DM1 by using functional magnetic resonance imaging (fMRI). Two motor task-evoked fMRI studies demonstrated higher activation in the sensorimotor network, including motor control areas, in DM1 compared to controls, although no correlations between changes of fMRI activation in the sensorimotor network and clinical characteristics in motor performance have been reported^13,14^. This lack of correlation may be attributed to several factors, including a lack of sensitivity of task-evoked fMRI or possible alterations in resting-state functional neuronal networks. Resting-state fMRI (rs-fMRI) is a functional brain imaging technique that can be used to evaluate spontaneous neuronal activity of the brain at rest by measuring low-frequency fluctuation of the blood oxygen level-dependent (BOLD) signal^15,16^. One way to analyze rs-fMRI data is to estimate functional connectivity (FC), which measures temporal synchronization of low frequency fluctuation between spatially distinct brain regions. A recent rs-fMRI study showed an increase in FC in the default mode network in patients with DM1 compared to controls^17^. Changes in FC and atypical personality traits were correlated in patients with DM1. More recently, graph theoretical analysis of rs-fMRI showed that patients with DM1 had reduced FC in a large frontoparietal network that correlated with impairment in visuospatial reasoning^18^. However, to the best of our knowledge, no studies have investigated functional alteration in the sensorimotor network in the resting brain, although motor dysfunction is the hallmark of clinical symptoms in DM1.

The resting-state power spectral density (PSD) approach, which measures the total power of a given time course within a specific frequency range (e.g., 0.01–0.08 Hz), has an advantage over the FC approach in that regional properties of the brain’s intrinsic functional dynamics can be estimated^19^. Power analysis of low-frequency fluctuation has been used to examine local spontaneous patterns during rest and has been applied in various neurological and psychological diseases^20–23^. Although the neurophysiological role of rs-PSD is not fully understood, there is growing evidence that PSD relates to baseline neuronal activity and long-range neural synchronization^16,24,25^.

In the current study, we aimed to investigate functional alterations in the resting-state sensorimotor network in patients with DM1 using PSD analysis of rs-fMRI data. We hypothesized that motor disability in DM1 is not an isolated deterioration of motor power but a multimodal dysfunction that also involves the visual system. We also assessed the relationship between motor-related clinical parameters and power spectral density in DM1. We further aimed to evaluate the possible involvement of white matter structures involved in the wiring of the motor network using PSD analysis of rs-fMRI data. For this second aim, we hypothesized that PSD of low frequency fluctuation in rs-fMRI would show abnormality in gray as well as white matter tissues.

## Results

### Demographic and Clinical Characteristics of Participants

The demographic and clinical characteristics are presented in Table 1. There were no significant differences for age and gender between healthy controls and patients with DM1 (*P* = 0.42 and *P* = 0.48, respectively).

**Table 1:** Demographic and clinical characteristics of study subjects.

	DM1 patients	Healthy controls	Statistics
	n = 18	n = 20	p-value
Age (year)	44.44 ± 10.66	45.70 ± 9.76	0.42
Gender (M:male,F:female)	M = 8, F = 10	M = 9, F = 11	0.48
Age onset (year)	29.58 ± 3.28		
Disease duration (year)	13.41 ± 2.02		
CTG repeat expansion	373.75 ± 66.30		
CK level	194.25 ± 26.25		
MRCSS	50.00 ± 2.82		
6 MWT (m)	375.34 ± 33.17		
LVEF	0.60 ± 0.01		
Mean ± SD			

Abbreviations: CK; creatine kinase, MRCSS; Medical Research Council sum score, 6 MWT; 6 minute walk test, LVEF; Left ventricle ejection fraction.

### Power spectral density group analyses

**Figure 1:**
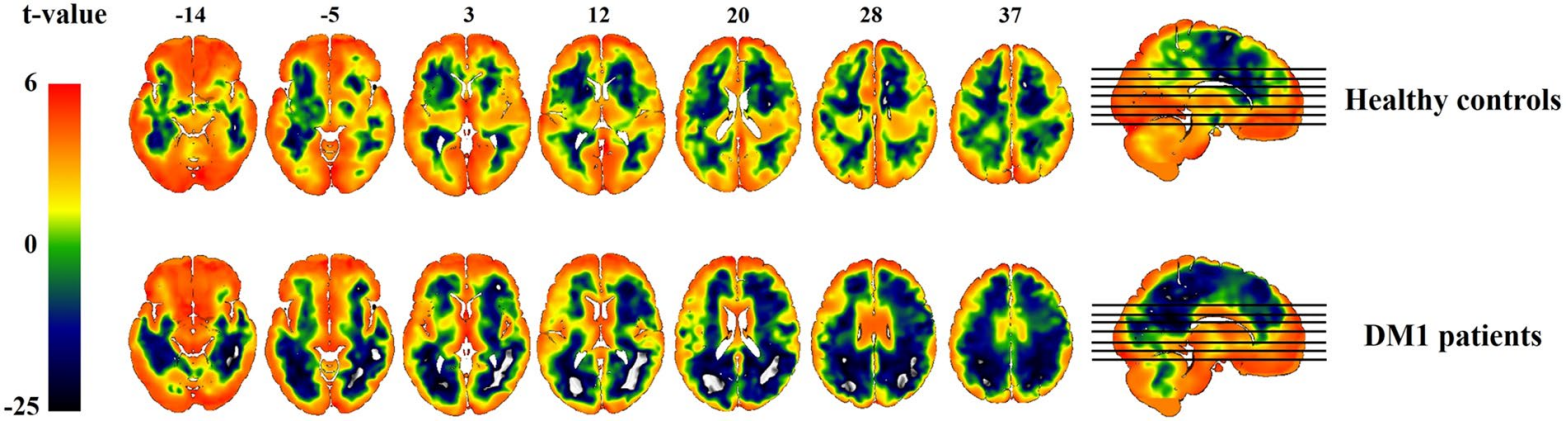
Power spectral density (PSD) map of spontaneous low frequency (0.001 Hz–0.01 Hz) oscillations in the resting brain of the patients with myotonic dystrophy type 1 (DM1) (Left) and healthy controls (Right).

**Figure 2:**
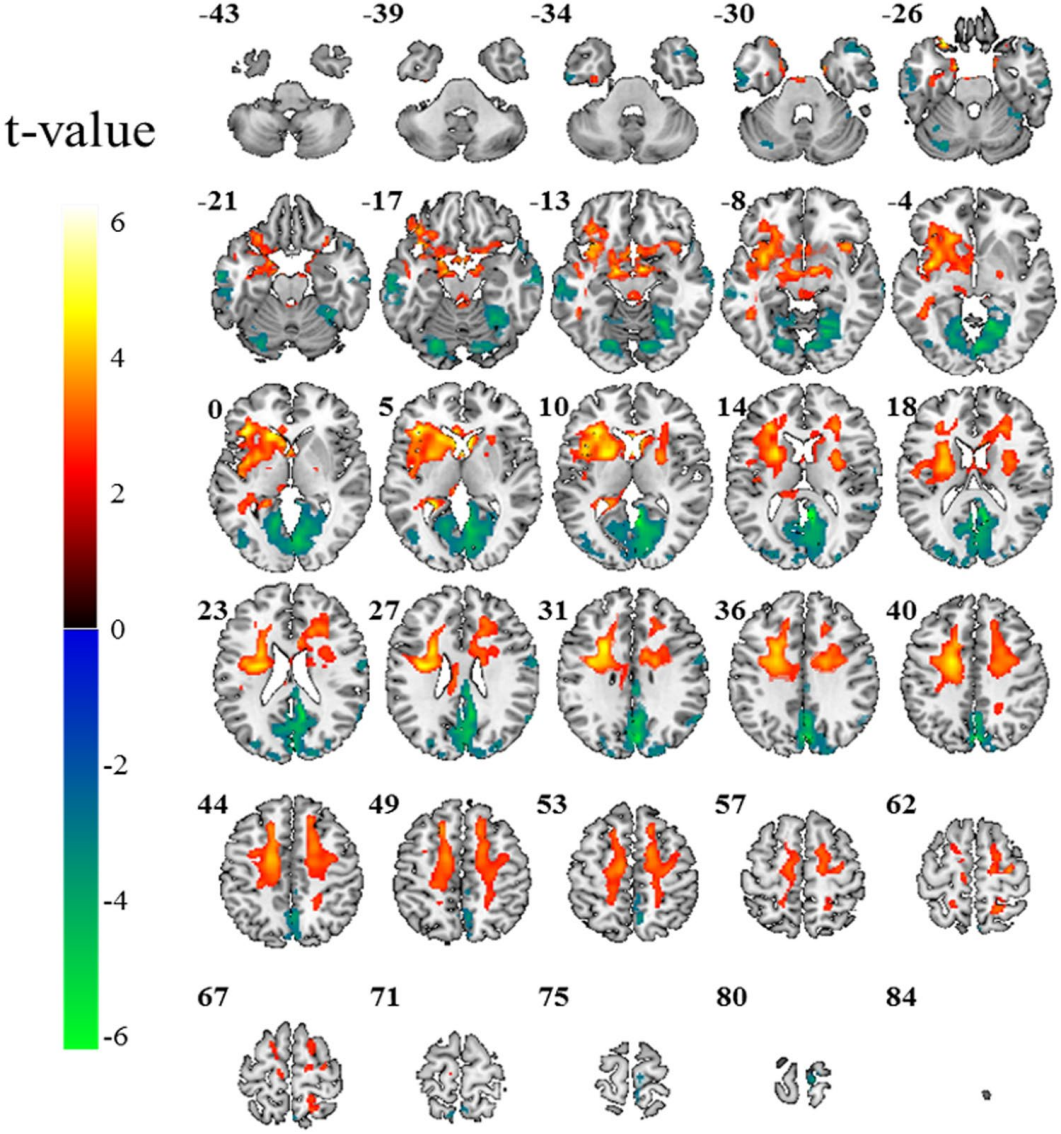
Group differences illustrated in brain power spectral density of low frequency (0.001 Hz–0.01 Hz) fluctuations. The red-yellow colors show brain areas with higher power in patients with DM1 than healthy controls and the blue-green colors show brain areas with lower power in patients with DM1 than healthy controls (corrected *P* < 0.05).

**Figure 3:**
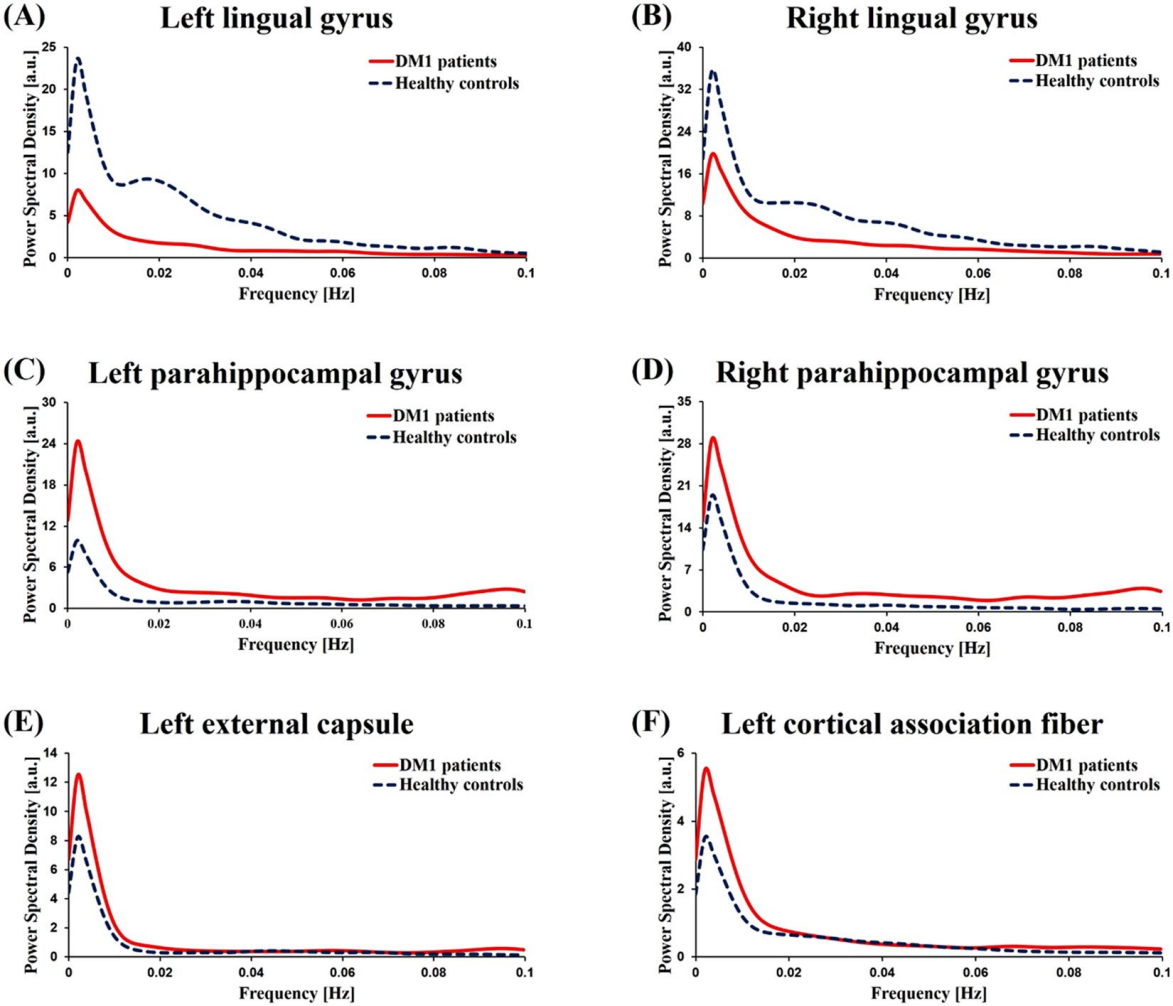
Group mean power spectral density of left lingual gyrus (**A**), right lingual gyrus (**B**), left parahippocampal gyrus (**C**), right parahippocampal gyrus (**D**), left external capsule (**E**), left cortical association fiber (**F**). In the patients with DM1, the PSDs in the bilateral parahippocampal gyrus, left external capsule and left cortical association fiber were significantly higher than the PSDs of healthy controls (*P* < 0.05). The PSDs in the bilateral lingual gyrus were significantly lower than the PSDs of healthy controls (*P* < 0.05).

The group power spectral density map of the patients with DM1 and healthy controls from the one sample t-test are shown in Fig. 1. The patients with DM1 showed higher power spectral density mostly in the frontal brain regions and lower power spectral density mostly in the occipital-parietal brain regions compared to healthy controls (corrected *P* < 0.05). For direct comparisons between the patients with DM1 and healthy controls using two-sample t-test, the patients with DM1 showed widespread power decrease in the posterior brain areas and power increase in the frontal brain areas compared to healthy controls (Table 2 and Fig. 2, corrected *P* < 0.05). Specifically, the middle and inferior temporal gyrus, postcentral gyrus, occipital gyrus, precuneus, posterior cingulate, and cerebellum showed power decrease in both hemispheres of patients with DM1. The superior temporal pole showed power decrease in the right hemisphere (Fig. 2 and Fig. 3). The orbitofrontal cortex, putamen, parahippocampal gyrus, fusiform gyrus, anterior insula cortex, and pallidum showed power increase in patients with DM1 compared to healthy controls (Figs 2 and 3).

**Table 2:** Group differences displayed in brain power spectral density of low frequency (0.001 Hz–0.01 Hz) fluctuations (corrected P < 0.05).

group	Region		Cluster size	Coordinates (mm)			Peak T
				x	y	z	
Patients with DM1	Cortical	L	10569	−20	−4	40	5.18
	association fiber	R	4707	20	6	44	3.97
>	External capsule	L	2271	−28	−8	20	5.13
	Putamen	L	579	−24	6	10	5.41
		R	185	24	2	10	4.29
Healthy controls	Anterior Insula	L	1255	−42	6	8	4.75
	cortex	R	147	38	16	−10	4.59
	Pallidum	L	97	−14	−2	−6	4.12
	Parahippocampal	L	209	−14	−8	−20	4.85
	gyrus	R	55	14	−6	−20	4.14
	Orbitofrontal cortex	L	517	−28	22	−24	5.66
	Fusiform gyrus	L	21	−38	−14	−24	3.76
Patients with DM1	Precuneus	R	502	4	−78	36	6.38
	Posterior cingulate	R	739	6	−52	12	9.18
<	Occipital gyrus	R	931	12	−80	10	4.77
Healthy controls	Middle temporal	L	235	−62	−26	−16	5.3
	gyrus	R	27	64	−8	−14	4.41
	Cerebellum	L	115	−24	−76	−18	5.12
		R	121	20	−76	−16	4.17

L: left, R: right.

The two-sample group difference map and group mean power spectral density (Figs 2 and 3) also showed that patients with DM1 had increased PSD in white matter (WM) structures, which are associated with motor function, compared to healthy controls. The WM structures showing increased PSD were the cerebral peduncle, head of caudate nucleus, anterior and posterior limb of internal capsule, external capsule, and cortical association fibers (corrected *P* < 0.05).

### Correlation analyses between clinical variables and Z-scores in the patients with DM1

**Figure 4:**
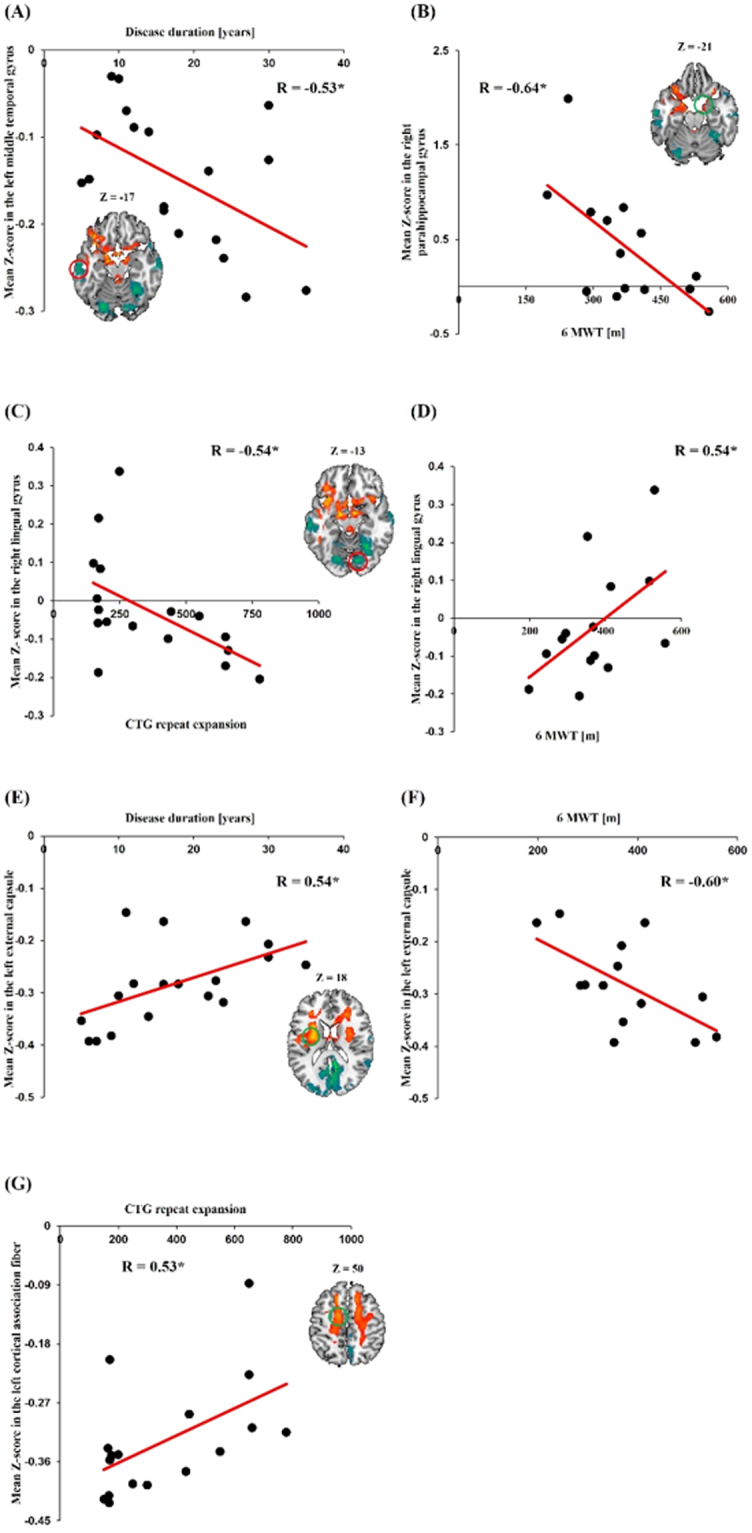
Correlation between mean Z-score related with power spectral density and clinical characteristics. The mean Z-score in the left middle temporal gyrus showed negative correlation with disease duration (**A**). The mean Z-score in the right parahippocampal gyrus showed negative correlation with the 6MWT (**B**). The mean Z-score in the right lingual gyrus showed negative correlation with CTG repeat expansion (**C**) and positive correlation with the 6MWT (**D**). The mean Z-score in the left external capsule showed positive correlation with disease duration (**E**) and negative correlation with the 6MWT (**F**). The mean Z-score in the left cortical association fiber showed positive correlation with CTG repeat expansion (**G**). (*P < 0.05).

In order to identify relationships between clinical variables and power spectral density in the patients with DM1, we performed correlation analyses. The patients with DM1 showed significant negative correlation between power in the left middle temporal gyrus and disease duration (r = −0.53, *P* = 0.024, Fig. 4A). The power of the right parahippocampal gyrus showed significant negative correlation with the 6MWT (r = −0.64, *P* = 0.013, Fig. 4B). The power of the right lingual gyrus was negatively correlated with CTG repeat expansion (r = −0.54, *P* = 0.026, Fig. 4C) and positively correlated with the 6MWT (r = 0.54, *P* = 0.048, Fig. 4D). With regard to white matter, in patients with DM1, the power of the left external capsule was positively correlated with disease duration (r = 0.54, *P* = 0.020, Fig. 4E) and negatively correlated with the 6MWT (r = −0.60, *P* = 0.023, Fig. 4F). The power of cortical association fibers was positively correlated with CTG repeat expansion (r = 0.53, *P* = 0.023, Fig. 4G).

## Discussion

In the current study, using the resting-state PSD method, we found that compared to healthy controls, patients with DM1 showed higher PSD responses in gray matter structures associated with visuospatial processing. Furthermore, patients with DM1 showed higher PSD responses in white matter structures associated with motor function. Correlation analysis indicated that the brain regions showing PSD differences were correlated with measures of motor performance.

One of the most interesting findings of our study is that patients with DM1 showed alteration in PSD in the brain regions strongly associated with visuospatial processing compared to healthy controls. Specifically, patients with DM1 showed reduced PSD in the lingual gyrus, occipital gyrus, middle temporal gyrus, and posterior cingulate area. The PSD of the right lingual gyrus, which is known to play an important role in visual memory and visual attention^26^, was positively correlated with the 6MWT in patients with DM1. These findings suggest that hypoactivity in the lingual gyrus is associated with visual attention as well as motor performance and, thus, suggests a relationship between visual attention and motor disability in DM1. Furthermore, the PSD of the right lingual gyrus was negatively correlated with CTG repeats. As CTG expansion is known to correlate with disease severity^1^, this negative correlation suggests that hypoactivity in the lingual gyrus is associated with disease severity. Imaging evidence has shown impaired visual and spatial function in DM1, and a recent imaging study on DM1 measured the cortical thickness in gray matter and found reduced volumes in parieto-temporo-occipital areas, which is consistent with visual and spatial dysfunction in DM1^27,28^.

The brain regions in which patients with DM1 showed increased PSD were the orbitofrontal cortex and parahippocampal area. The orbitofrontal cortex receives visual information from the occipital area via the inferior temporal lobe and reinforces the received stimuli^29^. Increased power in this area suggests that the received visual stimuli are hyper-reinforced. In addition, the PSD of the parahippocampal area showed a negative correlation with the 6MWT. The parahippocampal gyrus and its radiation to the posterior cingulate and retrosplenial areas have an important role in executive and visual processing^30^. Therefore, as in the lingual gyrus, these findings suggest that hyperactivity in the parahippocampal gyrus is associated with visual processing as well as motor performance in DM1. Finally, from our findings that the PSD is either reduced or increased in brain regions important in visual processing and its association with motor performance, we speculate that motor disability in DM1 is strongly associated with abnormality in the visual processing network.

Another major finding of our study is that WM structures associated with motor function showed increased baseline neuronal activity in DM1. Patients with DM1 showed increased PSD in the cerebral peduncle, basal ganglia, and cortical association fibers compared to healthy controls. Previously, several studies using diffusion tensor imaging (DTI) demonstrated structural abnormalities in the WM in patients with DM1^6–10^. However, to the best of our knowledge, our findings provide the first evidence for abnormal baseline neuronal activity of WM in patients with DM1. Among WM structures, the cerebral peduncles, through which the cortico-spinal, cortico-pontine, and cortico-bulbar tracts run through, play important roles in refining motor movements, learning of new motor skills, and converting proprioceptive information into balance and posture maintenance^31,32^. At the basal ganglia level, the head of the caudate nucleus, anterior and posterior limb of internal capsule, and external capsule showed increased PSD in DM1. The head of the caudate nucleus is involved in multimodal information processing and inhibition^33^. Via inhibition, the caudate nucleus is known to exert modulatory effects on motor activity and aid in the maintenance of selective motoric attention^34^. Anatomically, the posterior limb of the internal capsule is a key structure in the corticospinal tract and the anterior limb of the internal capsule is an important WM structure in the frontopontine tract; which originates from motor and non-motor frontal areas of the cerebral cortex and terminates in the pons, and is known to play a role in the coordination and regulation of movement^35^. The external capsule contains corticostriatal projection fibers connecting (pre)-frontal and temporal areas with the basal ganglia, which is known to play a major role in motion planning and execution. The PSD of the external capsule showed a negative correlation with the 6MWT. Taken together, our findings suggest that the motor deficits in DM1 involve both WM structures important for motor movement and for the coordination and regulation of movement.

Importantly, our findings demonstrated that our method of analysis with PSD is sensitive enough to detect subtle functional differences in WM between patients with DM1 and healthy controls. Although the findings in the white matter are not a standard when considering that PSD is higher in gray matter than in white matter^36^, we speculate that the sensitivity of PSD method in WM structures may come from the big differences in PSD of white matter structures between patients with DM1 and healthy controls. Furthermore, our findings regarding WM seem to add new information on widespread WM abnormalities found in patients with DM1. That is, in addition to structural WM abnormalities often measured with fractional anisotropy using DTI, our power spectral analysis revealed functional WM abnormalities in terms of spontaneous baseline neural activity in WM structures.

Our study is not without limitations. The small number of patients enrolled is a limitation that should be mentioned. Because the number of patients enrolled is too small, it would be necessary to perform the study with a large cohort of patients to draw general conclusions. However, with relatively few patients, we found a noteworthy significant difference. Most of the patients enrolled were ambulatory and our findings are not representative of patients with marked disease progression. Another major study limitation is the possible correlations between imaging findings and the neuropsychological or neuropsychiatric profile of the patients. Although the current study mainly focused on the alteration of the sensorimotor resting state network, the correlations between imaging findings and the neuropsychological profile would provide valuable information. Therefore, the further study on the possible correlations between power spectral density and the neuropsychological or neuropsychiatric profile of the patients is warranted. Furthermore, this was a cross-sectional study; thus, caution must be exerted in interpreting the results and longitudinal studies are warranted for further clarification^37^.

## Conclusion

In the current study, we found that patients with DM1 showed widespread PSD alteration in gray and white matter. In gray matter, we found that the PSD in patients with DM1 is either reduced or increased in brain regions important for visual processing and that PSD in these regions was associated with motor performance. These findings, therefore, strongly suggest that motor disability in DM1 is not an isolated deterioration of motor power but a multimodal dysfunction that also involves the visual system. We also found that WM structures associated with motor function showed increased PSD in patients with DM1. The widespread PSD alteration in these WM structures suggests that motor deficits in DM1 involve both WM structures important for motor movement and for its coordination and regulation.

## Methods

### Participants

The participants included 18 patients with adult onset DM1 (8 men; 10 women) and 20 healthy controls (9 men; 11 women). The mean age of the patients was 44.44 (SD = 10.66) years. The past medical history of all patients enrolled was reviewed and they had no previous record of medication that could affect hyper-somnolence state in these patients. Patients with DM1 were age-, gender-, and education level- matched to healthy controls (mean age = 45.70, SD = 9.76 years). Clinical characteristics, disease duration, CTG repeat expansion, creatine kinase levels, motor power, 6-minute walk test (6MWT) findings, and laboratory findings were recorded. Written informed consent was obtained from each participant in accordance with the requirements of the Kyungpook National University Hospital Human Research Committee. All procedures were approved by the Kyungpook National University Institutional Review Board.

### Motor function evaluation

The motor function of the patients was evaluated by the Medical Research Council (MRC) sum score and 6MWT. The MRC sum score (MRCSS) is calculated by summation of the MRC scores of six muscle groups (maximum score is 60), including shoulder abduction, elbow flexion, extension of the wrist, hip flexion, extension of the knee, and dorsiflexion of the foot on both sides. We also evaluated the MRC of handgrip and ankle plantar flexion. In addition, the 6MWT was performed and the results were measured.

### Genetic study

Genomic DNA was isolated from peripheral blood, with consent from each individual, using the Wizard genomic DNA purification kit (Promega, Madison, WI). Polymerase chain reaction (PCR) with primers DM1-F and DM1-R was used to amplify the region of the *DMPK* gene including the CTG repeat. To estimate allele size, the GeneScan analysis program on an automated sequencer (ABI Prism 3130 Genetic Analyzer, Applied Biosystems) was used. Southern blot analysis was performed to detect the larger allele of the CTG expansion.

### Resting state BOLD fMRI data acquisition

Resting-state BOLD fMRI data was collected for each participant using a 3 T scanner (Discovery MR750, GE healthcare) at Kyungpook National University Medical Center, Korea. A thirty-two channel head coil was used for image acquisition. During resting-state fMRI data acquisition, the participants had no other task but to stay alert and keep their eyes closed. In the resting-state fMRI scan, 240 volumes were acquired using T2*-weighted echo planar imaging (EPI). Acquisition parameters were as follows: repetition time (TR) = 2000 ms, echo time (TE) = 30 ms, field of view (FOV) = 21 cm, acquisition matrix = 64 × 64, slice thickness = 4 mm, and flip angle = 90°. Structural brain images were acquired using 3D Brain volume imaging (BRAVO) sequence for high-resolution T1-weighted anatomical images (TR = 8.16 ms, TE = 3.18 ms, 1-mm iso-voxel resolution).

### Resting state BOLD fMRI data preprocessing

The resting-state fMRI data were preprocessed with the statistical parametric mapping software SPM 8 (http://www.fil.ion.ucl.ac.uk/spm/), including slice-timing, realignment, normalization into a Montreal Neurological Institute template based on the standard stereotaxic coordinate system, and spatial smoothing with an 8 mm (full-width at half-maximum) Gaussian kernel. Resting-state fMRI data were then prepared for analysis of power spectral density of spontaneous low-frequency BOLD fluctuation.

### Power spectral density analysis and group analyses

Preprocessed data were used to create a power spectral density map. The signal was extracted from each voxel, and it was calculated to estimate the power spectral density at the low frequency band (0.001 Hz–0.01 Hz) using Welch’s method in Matlab (MathWorks, Inc., Natick, MA)^16,38,39^. To standardize the power raw measures, Z-transform was performed for the power spectral density map across the whole brain as follows:

$$Z=(\frac{x-m}{s}),\,m=\frac{1}{N}\sum _{i=1}^{N}{x}_{i},\,s=\sqrt{\frac{1}{N}\sum _{i=1}^{N}{({x}_{i}-m)}^{2}}$$

where $x$ is the power value, $N$ is the number of voxels within the whole brain, $m$ represents the global mean across the whole brain, and $s$ is the global standard deviation^16^.

Using the Z-score map, a one-sample t-test was performed within each group and a two-sample t-test was performed to evaluate the power difference between the two groups. Voxels with a *P*-value < 0.005 and cluster size >5 voxels were considered as showing a significant difference between the two groups, which was equal to a corrected threshold of *P* < 0.05, determined by Monte Carlo simulation using AlphaSim as implemented in the SPM REST toolbox^40^. Input parameters to AlphaSim included an individual voxel threshold probability of 0.005, cluster connection radius of 5 mm, and 8 mm full width at half maximum smoothness. The estimated minimum cluster size extent was 52 voxels for the two-sample t-test map in order to satisfy a family-wise error rate correction of *P* < 0.05.

### Statistical analysis

Pearson correlation analyses were used to determine the correlations between clinical characteristics and the Z-score of the power spectral density in the patients. All statistical analyses were performed using the SPSS software (SPSS, Inc., Chicago, IL, v. 20) and statistical significance was set at *P* < 0.05.

### Data availability

The datasets generated during and/or analysed during the current study are available from the corresponding author on reasonable request.
